# Photoreversible Patterning of Biomolecules within Click-Based Hydrogels**

**DOI:** 10.1002/anie.201106463

**Published:** 2011-12-08

**Authors:** Cole A DeForest, Kristi S Anseth

**Affiliations:** * Chemical & Biological Engineering, University of Colorado and the Howard Hughes Medical Institute424 UCB, Boulder, CO 80309-0424 (USA)http://www.colorado.edu/che/ansethgroup/; ** We thank Dr. A. Kloxin and M. Tibbitt for useful discussions on photopatterning. Fellowship assistance to C.A.D. was awarded by the US Department of Education Graduate Assistantships in Areas of National Need. This work was supported financially by the National Science Foundation(DMR 1006711) and the Howard Hughes Medical Institute.

**Keywords:** bioorganic chemistry, hydrogels, peptides, photochemistry, RGD peptides

Polymer-based hydrogels have emerged as a unique class of biomaterials that enable cells to be cultured in three dimensions within user-defined synthetic microenvironments.^1^–^3^ Based on the specific hydrogel formulation, the material properties of cell-laden constructs can be precisely defined to impart different moduli, chemical moieties, porosity, adhesivity, degradability, and stimuli responsiveness over nano, micro, and macroscopic scales. Cells have been engineered to proliferate within, migrate through, and undergo differentiation inside these materials by tuning the initial properties of these networks through the incorporation of physiologically relevant cues.^4^

More recently, hydrogel platforms that permit the introduction of biochemical epitopes at any point in time and space to affect cell function dynamically after encapsulation have been developed.^5^ Although these techniques have been successfully utilized to control cell adhesion and motility,^6^, ^7^ promote endothelial tubulogenesis,^8^, ^9^ and direct cell outgrowth,^10^, ^11^ complementary platforms that enable the introduction and subsequent removal of these signals would be beneficial. For example, such systems would allow the dynamic presentation of signaling biomolecules that are found in the native, temporally variable niche occupied by stem cells to be recapitulated more closely. Herein, we demonstrate that the combination of two bioorthogonal photochemical reactions enables the reversible spatial presentation of a biological cue, as well as the formation of complex, well-defined, biomolecular gradients within a hydrogel. The results of this study highlight how the regulation of the biochemical environment can be used in the development of more sophisticated cell culture substrates.

The reversible patterning strategy is based on the combination of two orthogonal, biocompatible photoreactions.^11^ The thiol-ene reaction, which involves the radical-mediated addition of a thiol to an alkene, is readily initiated by visible light (*λ*=490–650 nm) and an appropriate photoinitiator (eosin Y).^12^, ^13^ The second reaction is the photoscission of an *o*-nitrobenzyl ether to give a nitroso compound and an acid by-product upon exposure to UV light (*λ*=365 nm).^14^, ^15^ By synthesizing the biological molecule of interest to contain both the thiol group for the photocoupling reaction and the photolabile *o*-nitrobenzyl moiety (Figure 1 a), the thiol-ene and photocleavage reactions can be used to attach and subsequently remove covalently bound bioepitopes in hydrogel networks, respectively (Figure 1 b). As these reactions arephotomediated, both the introduction and subsequent removal of relevant biomolecules can be explicitly controlled in space and time by exposure to light.

**Figure 1:**
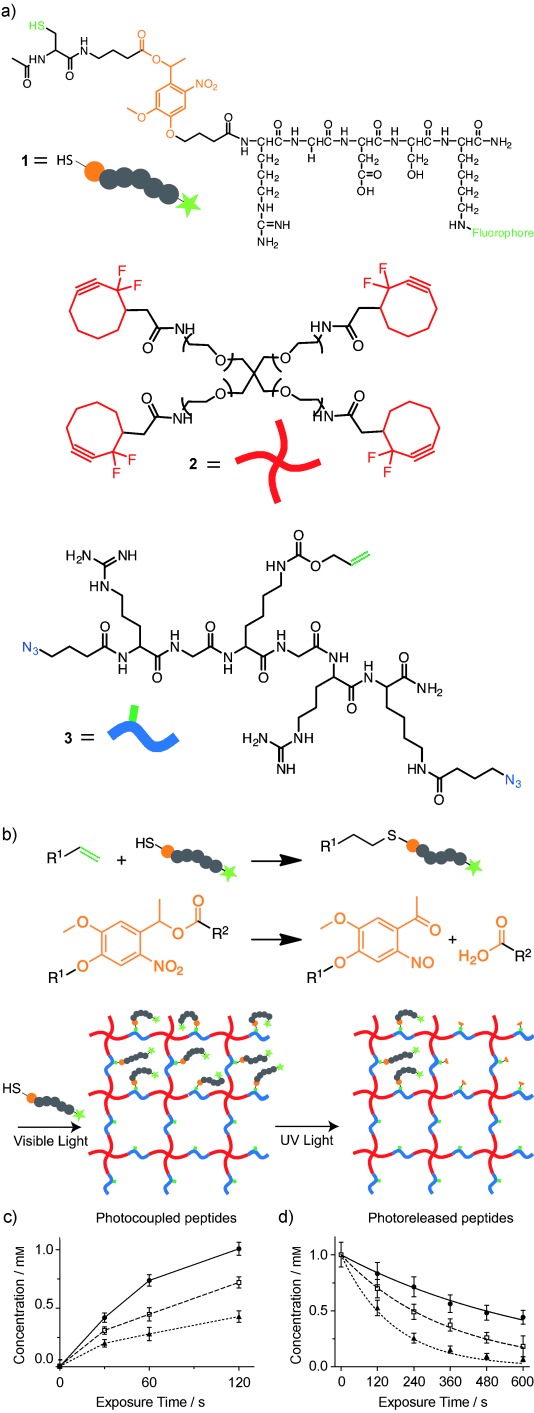
a) Hydrogels were formed from PEG tetracyclooctyne (*M*_n_=ca. 10 000 Da, (**2**)) and N_3_-RGK(alloc)GRK(N_3_)-NH_2_ (**3**). The alloc groups were functionalized with the fluorescent peptide Ac-C-(PL)-RGDSK(AF_488_)-NH_2_ (**1**) through thiol-ene photoreaction and unfunctionalized through *o*-nitrobenzyl ether photocleavage. b) Representation of the thiol-ene conjugation reaction (top), the *o*-nitrobenzyl ether cleavage reaction (middle), and the overall hydrogel structure and reversible patterning approach (bottom). c) Concentrations of peptide **1** patterned by thiol-ene reaction as a function of the initiator concentration (▴=2.5 μm, □=5 μm, •=10 μm) and visible light exposure time. d) Experimentally-determined and predicted concentrations (lines) of **1** based on the photocleavage kinetics from Eq. (1) as a function of UV light intensity (•=5 mW cm^−2^, □=10 mW cm^−2^, ▴=20 mW cm^−2^) and exposure time.

The multifunctional patterning peptide was synthesized by a combination of standard Fmoc solid-phase methods, in which the photodegradable azide acid 4-(4-(1-(4-azidobutanoyloxy)ethyl)-2-methoxy-5-nitrophenoxy)butanoic acid^11^ was coupled to the N-terminus of the peptide sequence of interest. The terminal azide moiety served as a protecting group during peptide synthesis, which ensured that only one photodegradable moiety was present per peptide. This azide group was readily reduced on the resin by the Staudinger reaction^16^ with triphenylphosphine, which liberated the N-terminal primary amine for further peptide synthesis. The photoreversible patterning agent Ac-C-(PL)-RGDSK-(AF_488_)-NH_2_ (**1**, AF_488_=Alexa Fluor 488), which is based on the ubiquitous cell-adhesion ligand RGD, was prepared and contained both a photoreactive thiol on the cysteine residue, and an adjacent photodegradable *o*-nitrobenzyl ether moiety (PL).

The hydrogel was formed by the copper-free, strain-promoted, azide–alkyne cyclooaddition (SPAAC) click reaction between poly(ethylene glycol) (PEG), tetracyclooctyne^17^, ^18^ (**2**), and bis(azido), allyloxycarbonyl (alloc)-protected polypeptide N_3_-RGK(alloc)GRK-N_3_ (**3**) in an aqueous medium (Figure 1 a).^7^, ^11^, ^19^ The resulting idealized SPAAC-based network is homogenously populated with alloc functionalities that contain photoreactive alkenes. These alkenes serve as anchor points for the introduction of biochemical cues by the thiol-ene photoconjugation reaction. As a side note, this reaction is fully cytocompatible,^7^, ^20^ which means that cells can be readily encapsulated and cultured in these gels.

Peptide **1** was swelled into the hydrogel network and the degree of thiol-ene photoconjugation was controlled by varying the exposure time of the irradiation with visible light (10 mW cm^−2^, 0–120 s) or the concentration of the eosin Y (2.5, 5, or 10 μm, Figure 1 c). Eosin Y absorbs light between *λ*=450 and 550 nm, with a maximum absorbance at approximately 515 nm. The patterned peptide concentrations ranged from 0–1.0 mm. This concentration range is biologically relevant as numerous groups have used concentrations of peptides that range from mm–nm to manipulate the binding of integrins or to sequester growth factors, which ultimately directs critical cellular functions.^5^, ^21^ Subsequently, gels that contained approximately 1.0 mm of patterned Ac-C-(PL)-RGDSK(AF_488_)-NH_2_ (**1**) were exposed to UV light at different intensities (5, 10, or 20 mW cm^−2^) for various time periods (0–600 s) to cleave the *o*-nitrobenzyl ether group, which absorbs strongly between *λ*=325–415 nm, and photorelease the patterned peptide (Figure 1 d). As the photodegradable *o*-nitrobenzyl ether moiety absorbs light and cleaves at wavelengths less than 415 nm, both the addition and cleavage photoreactions can be performed independently by irradiating the system with different light sources. Furthermore, each of these reactions can be initiated with multiphoton light (*λ*=860 nm for addition, 740 nm for cleavage), which offers the opportunity to independently control these reactions in 3D, as well as the ability to dynamically tune the pericellular region to assay how cells respond to biochemical changes in their local environment.

The photocleavage reaction was found to be a first order degradation with a rate constant (*k*) given by:

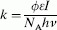
(1)

where *ϕ* is the quantum yield (0.020),^11^ *ε* is the molar absorptivity (4780 m^−1^ cm^−1^ at *λ*=365 nm),^11^ *I* is the intensity of the incident light, *N*_A_ is the Avogadro constant, *h* is the Planck constant, and *ν* is the frequency of light. The value of the rate constant indicates that the patterned peptide is almost fully removed (more than 97 %) after 10 min of irradiation with 20 mW cm^−2^ of UV light, or in real time with multiphoton light (*λ*=740 nm). This time scale is relevant for many typical cell-culture applications, such as the migration and differentiation of cells, in which cellular responses are on the order of several hours to days. From this analysis, the precise amount of peptide that is photoreleased can be readily predicted for a given light exposure (lines in Figure 1 d).

As well as controlling the introduction and removal of functional groups in the network by varying the patterning conditions, spatial control of both the photoaddition and photoremoval reactions was achieved by selectively exposing subvolumes of the hydrogel to light. By using traditional photolithographic techniques, whereby the sample is exposed to masked light, a 2D pattern of shapes (200 μm lines spaced 200 μm apart) was transferred throughout the thickness of a gel. This pattern was then partially removed upon exposing the gel to UV light through a second photomask to obtain a series of checkered parallel lines (Figures 2 a, c). By focusing pulsed laser light within the hydrogel volume, the functionalization of the network was controlled in 3D to first create a double helix, and then to remove part of the structure to form a single helix (Figures 2 b, d). The patterning resolution was limited by the optical limits of the equipment to approximately 1 μm in the *x*-*y* plane for both photolithographic and multiphoton-based techniques, and to approximately 3–5 μm in the *z*-plane for the multiphoton technique. As these distances are smaller than a typical mammalian cell, these reactions should prove useful for many applications that are focused on modifying the local microenvironment around individual encapsulated cells.

**Figure 2:**
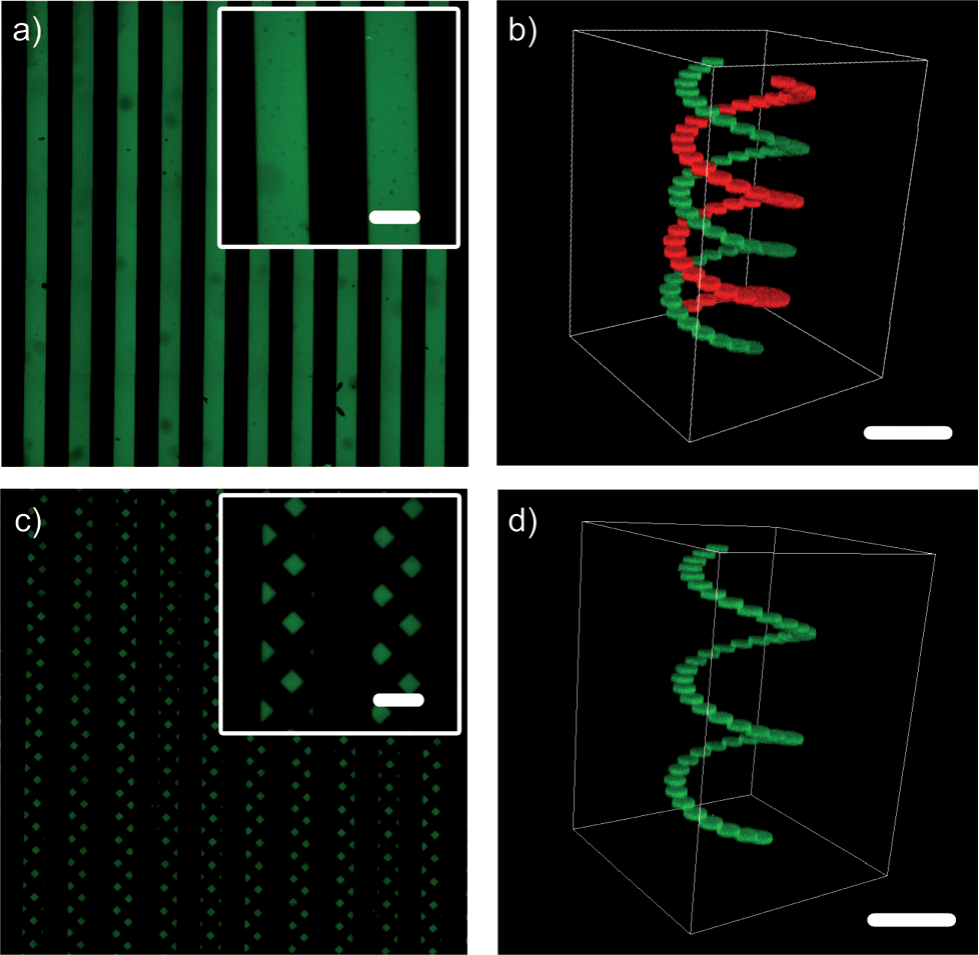
Thiol-ene patterning of the fluorescent peptide Ac-C-(PL)-RGDSK(AF_488_)-NH_2_ (**1**) into the hydrogel a) in 2D throughout the gel after exposure to masked visible light and b) in 3D after exposure to focused pulsed laser light. False coloring is used for enhanced visualization. c) and d) Subsets of the pre-patterned cues were removed by exposure to UV light to modify the original chemical pattern and yield new 2D and 3D patterns. Scale bars=200 μm.

As well as the presentation of discrete signals, graded biochemical signaling cues are essential for many biological processes, and can be used to screen the effects of various morphogens rapidly, over a wide range of concentrations. Graded signaling can also be used to study chemotaxis and other phenomena. After patterning the gel with a fixed amount of **1** (ca. 1 mm) with visible light, non-linear gradients were formed within the material by exposing the patterned substrate to a gradient of UV light (10 mW cm^−2^). The gradient was generated with a moving opaque photomask^22^ that covered the sample at a rate of 0.4, 0.8, and 1.6 mm min^−1^. For continuous gradients of light exposure, this approach resulted in exponential gradients in the final concentration of **1** with predictable decay constants of 0.175 min^−1^/(rate of sample coverage). By photoreleasing peptides that were initially present in a uniform fashion throughout the bulk of the network (Figure 3 a, d) or within line patterns (Figure 3 c, e), intricate exponential gradients were formed over millimeter-scale distances within the material environment. These gradients were either continuous or discrete line patterns. Alternatively, by shuttering the light, more complex stepped gradients were formed, in which regions of constant peptide concentration were interspaced with regions of decreasing concentration (Figure 3 b). Each of these gradients was readily predicted for a given light exposure, and the predictions correlated well with the experimental findings.

**Figure 3:**
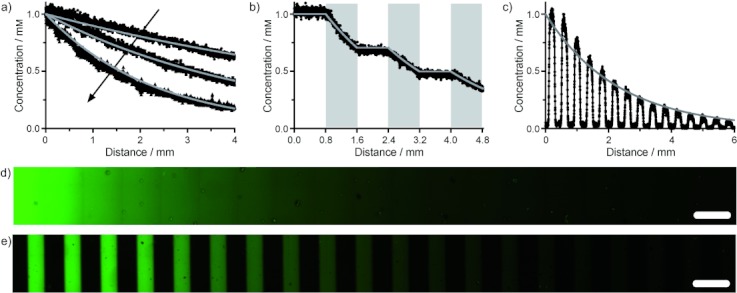
Ac-C-(PL)-RGDSK(AF_488_)-NH_2_ (**1**) was patterned into hydrogels by using unmasked light (panels (a), (b), and (d)) or a mask that contains 200 μm slits spaced 200 μm apart (panels (c), (e)). The prepatterned samples were subsequently exposed to gradients of UV light that were generated by a moving opaque photomask (1.6, 0.8, or 0.4 mm min^−1^ for (a); 0.4 mm min^−1^ for (b–e)) to induce photodegradation of the PL moiety and to create exponentially decaying peptide gradients. By shuttering the light (panel (b)) or releasing pre-patterned lines (panels (c), (e)), unique gradients in the peptide concentration were generated across the network. Solid gray lines are predicted concentrations based on predetermined photocleavage kinetics from Eq (1). Scale bars=400 μm.

Ultimately, this chemistry is useful as it can be performed in the presence of cells because the wavelengths, exposure times, and intensity of light are all cytocompatible.^11^, ^23^ To demonstrate that this patterning approach offers dynamic control over cell function, mouse embryonic fibroblast (NIH 3T3) cells were seeded at 8×10^3^ cells cm^−2^ onto constructs that contained patterned islands of **1** (ca. 1 mm). The initial attachment of the cells was confined to the adhesive regions of the material that was functionalized with **1** (200 μm lines of **1** spaced 200 μm apart, Figure 4 a). The cells only adhered to and exhibited a spread morphology on these regions. Twenty-four hours after seeding, the adhesive ligand was removed from selective areas of the initial pattern by rotating the same 200 μm line pattern by 45° clockwise and exposing the material to UV light. This procedure caused the cells within these user-defined regions to detach from the surface of the material within minutes of exposure (Figure 4 b). These cells were subsequently isolated, expanded, and further cultured. This method provides an efficient way of potentially sampling a subset of a total cell population for assaying the proliferation, gene expression, metabolic activity, and differentiation of stem cells within user-defined regions of the culture substrate.

**Figure 4:**
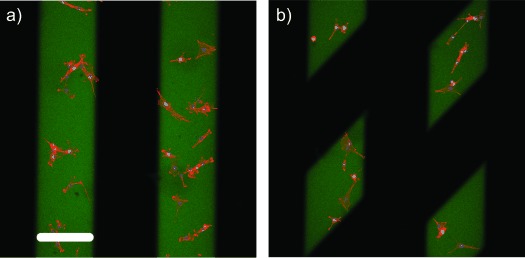
a) Parallel lines (200 μm wide) of Ac-C-(PL)-RGDSK(AF_488_)-NH_2_ (**1**) were patterned into the hydrogel at 1.0 mm by visible thiol-ene photocoupling. Seeded NIH 3T3 cells only adhered to regions of the hydrogel surface that were functionalized with **1**. b) Localized cell-detachment was observed upon spatial removal (24 hours post-seeding) of RGD through masked UV light exposure. Green=RGD (**1**), red=F-actin, blue=nuclei. Scale bar=200 μm.

By utilizing multiple, bioorthogonal, light-based reactions, we were able to exert spatiotemporal control over the reversible presentation of biologically relevant chemical cues. The level of control that can be obtained over the biochemical functionality of the hydrogel platform should enable dynamic cell functions to be assayed selectively, and for the cellular microenvironment to be programmed on demand. We expect such approaches for dynamically controlling the properties of materials to be helpful in establishing a more complete understanding of how cells respond to their extracellular environment, and assist in the rational design of cell-delivery systems for applications in regenerative medicine.

## Experimental Section

Complete experimental procedures for peptide and macromolecular precursor synthesis, formation of the network, photoreversible patterning, generation of the gradient, and cell culture, seeding, and visualization are given in the Supporting Information.

## Supporting Information

Detailed facts of importance to specialist readers are published as ”Supporting Information”. Such documents are peer-reviewed, but not copy-edited or typeset. They are made available as submitted by the authors.
