# Supplementary material for: Photoreversible Patterning of Biomolecules within Click-Based Hydrogels**
Source: Angew Chem Int Ed Engl. 2011 Dec 8;51(8):1816–9. doi: 10.1002/anie.201106463 (PMC3430005; doi:10.1002/anie.201106463)
Supplement: Supplementary file 1 [file anie0051-1816-SD1.pdf]

Angewandte Chemie
Eine Zeitschrift der Gesellschaft Deutscher Chemiker

Supporting Information

© Wiley-VCH 2012

69451 Weinheim, Germany

Photoreversible Patterning of Biomolecules within Click-Based Hydrogels**

*Cole A. DeForest and Kristi S. Anseth**

anie_201106463_sm_miscellaneous_information.pdf

Supporting Information

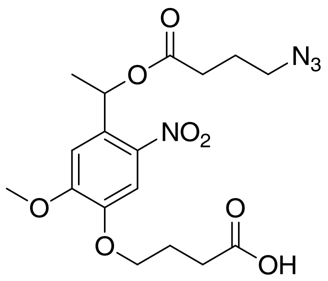

Synthesis of PLazide: The photolabile nitrobenzyl ether moiety containing flanking carboxylic acid and azide functionality (PLazide) was synthesized as previously described[11]. Briefly, 4-azidobutanoic acid was synthesized by the azidification and deprotection of ethyl-4-bromobutyrate, and its anhydride was reacted (5X/OH) overnight with 4-[4-(1-Hydroxyethyl)-2-methoxy-5-nitrophenoxy]butanoic acid (Hydroxyethyl photolinker, EMD Novabiochem) in minimal dichloromethane (DCM). After washing, the pure product (PLazide) was recovered by flash chromatography in excellent yield (92%) as a yellow solid. ^{1}H NMR (500 MHz, DMSO-d_6, δ): 12.18 (s, 1H), 7.57 (s, 1H), 7.10 (s, 1H), 6.21 (q, 1H), 4.07 (t, 2H), 3.93 (s, 3H), 3.32 (t, 2H), 2.4 (p, 4H), 1.95 (p, 2H), 1.76 (p, 2H), 1.58 (d, 3H); ^{13}C NMR (101 MHz, DMSO-d_6, δ): 173.98, 171.56, 153.53, 146.89, 139.66, 131.87, 108.66, 108.42, 67.94, 67.48, 56.25, 49.87, 30.70, 29.92, 23.98, 23.72, 21.30; HRMS (ESI, m/z): [M – ^{1}H]$^-$ calc for $C_{17}H_{21}N_4O_8^-$, 409.1368; found, 409.1363.

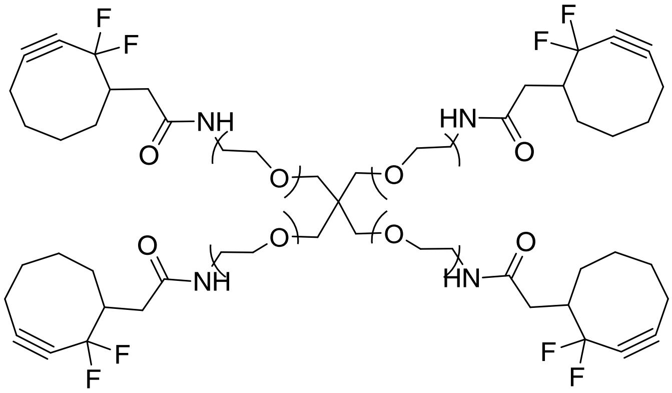

Synthesis of PEG tetraDIFO3: Difluorinated cyclooctyne (DIFO3) was synthesized from 1,3-cyclooctanedione[18] following a published synthetic route[17] and was reacted (1.5X/NH_2) overnight with 2-(1H-7-Azabenzotriazol-1-yl)--1,1,3,3-tetramethyl uronium hexafluorophosphate Methanaminium (HATU, 1.5X/NH_2) and 4-arm PEG tetraamine (M_n ~ 10,000 Da, JenKem) in minimal dimethylformamide (DMF). The crude product was concentrated by rotary evaporation, dissolved in dH_2O, dialyzed (MWCO ~ 2 kDa, SpectraPor) for 72 hrs, filtered, and lyophilized to give a white powder in excellent yield (96%). By comparing characteristic DIFO3 ^{1}H-NMR peaks with those from the PEG backbone, the percentage modification was confirmed to be >95%.

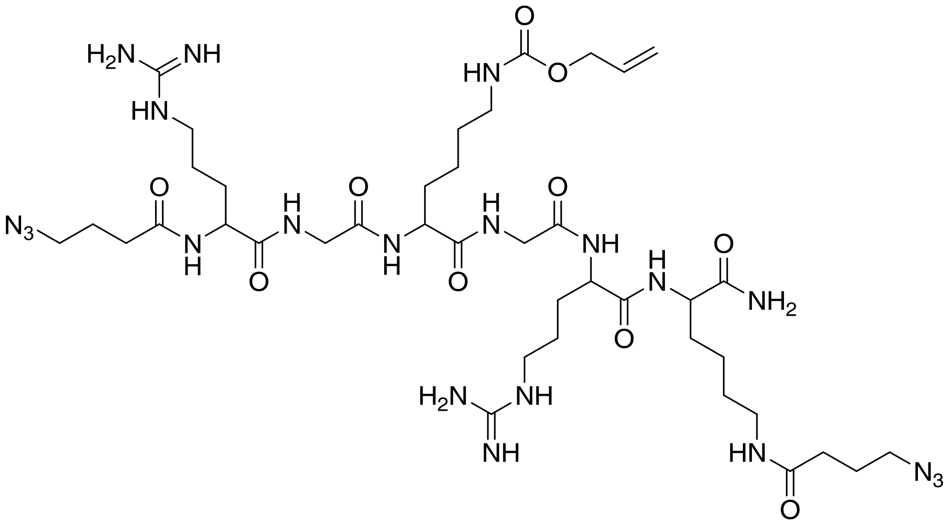

Synthesis of Peptide Crosslinker: The allyl ester-containing polypeptide H-RGK(alloc)GRK(Dde)-NH_2 was synthesized (Protein Technologies Tribute peptide synthesizer) *via* Fmoc solid-phase methodology and HATU activation. The 1-(4,4-dimethyl-2,6-dioxacyclohexylidene)ethyl (Dde) group was removed with 2% hydrazine monohydrate (Sigma) in DMF (3 x 10 min), and 4-azidobutanoic acid was coupled *via* standard HATU coupling chemistry to the N-terminus and the ε-amino group of the C-terminal lysine. Resin was treated with trifluoroacetic acid (TFA)/triisopropylsilane (TIPS)/water (95:2.5:2.5) for 2 hours, and precipitated in and washed with ice-cold diethyl ether (3x). Crude peptide was purified to give N_3-RGK(alloc)GRK(N_3)-NH_2 using semi-preparative RP-HPLC (Waters Delta Prep 4000) using a 70 minute linear gradient (5 to 95%) of acetonitrile and 0.1% TFA (Sigma), and purity was confirmed by analytical RP-HPLC and MALDI-TOF-MS: $[M + {}^1H]^+$ calc for $C_{40}H_{72}N_{21}O_{10}^+$, 1007.1; found, 1007.7.

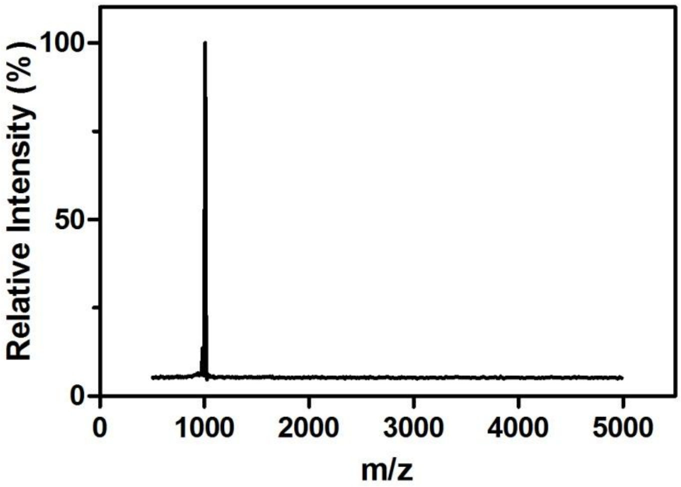

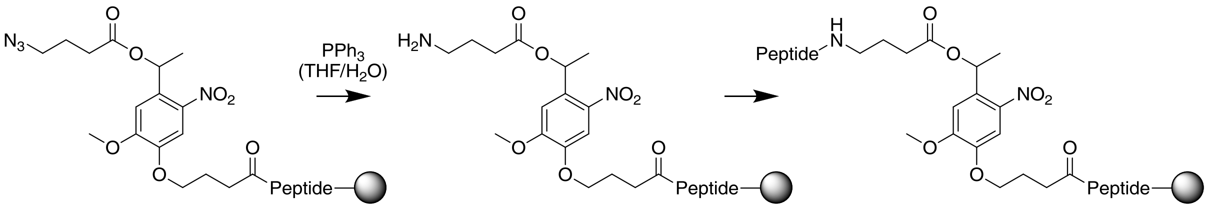

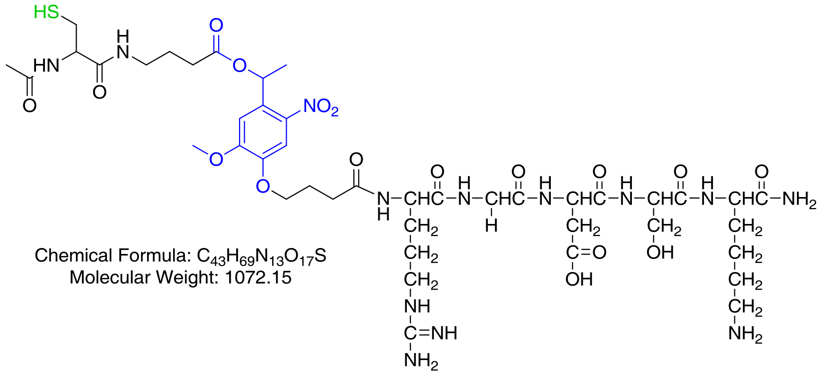

Synthesis of Photoreleasable Patterning Peptide (Ac-C-[PL]-RGDSK(AF_{488})-NH_2): The peptide H-RGDSK-NH_2 was synthesized *via* Fmoc solid-phase methodology and HATU activation, and PLazide was coupled to the N-terminal amine by standard HATU coupling chemistry. Resin was treated with 5 wt% triphenylphosphine in tetrahydrofuran (THF)/water (90:10) for 12 hours to reduce the terminal azido functionality to a primary amine to which Fmoc-Cys(Trt)-OH (Anaspec) was coupled. The terminal Fmoc functionality was cleaved (20% piperidine in DMF) and the peptide was capped with acetic anhydride. Resin was treated with TFA/TIPS/water (95:2.5:2.5) for 2 hours, and precipitated in and washed with ice-cold diethyl ether (3x). Crude peptide was purified to give Ac-C-[PL]-RGDSK-NH_2 using semi-preparative RP-HPLC using a 70 minute linear gradient (5 to 95%) of acetonitrile and 0.1% TFA. The product was lyophilized to give a yellow solid, and purity was confirmed by analytical RP-HPLC and MALDI-TOF-MS: $[M + {}^1H]^+$ calc for $C_{43}H_{70}N_{13}O_{17}S^+$, 1073.2; found, 1072.4. Ac-C-[PL]-RGDSK-NH_2 (20 mg) was dissolved in DMF (4 mL) containing Alexa Fluor® 488 carboxylic acid, 2,3,5,6-tetrafluorophenyl ester (1 mg) with N,N-Diisopropylethylamine (DIEA, 50 µL) and reacted overnight protected from light. The sample was concentrated, dissolved in dH_2O, dialyzed for 72 hrs (MWCO ~ 500 Da, SpectraPor), and lyophilized to give a yellow solid, denoted Ac-C-[PL]-RGDSK(AF_{488})-NH_2.

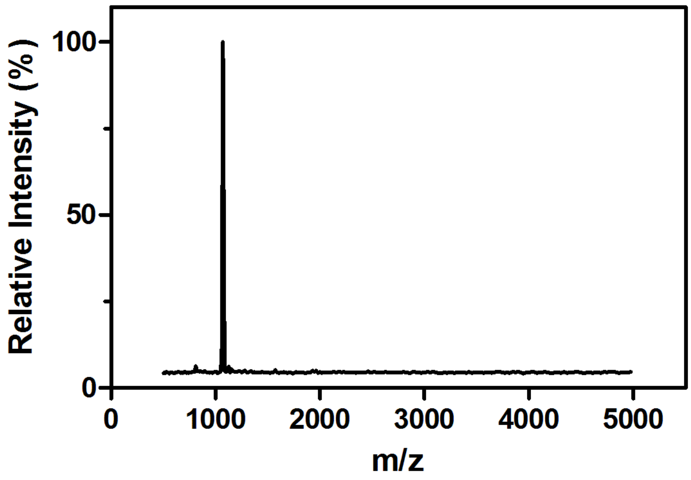

Relative Intensity (%)
100
50
0
0
1000
2000
3000
4000
5000
m/z

Formation of SPAAC-based Hydrogel: A 10 wt% solution of PEG tetraDIFO3 and N_3-RGK(alloc)GRK(N_3)-NH_2 was prepared in phosphate buffered saline (PBS) with equal concentrations of azide and alkyne functionalities. The macromer solution was reacted between azide-functionalized[11] and Rain-X®-treated glass slides separated with 1 mm thick gaskets for 1 hr. The slides were separated and gels were swollen overnight in PBS prior to usage.

Biological Patterning within Click-based Hydrogel: Click-based hydrogels were swollen for 1 hr in Ac-C-[PL]-RGDSK(AF_{488})-NH_2 (3 mg mL^{-1}) and eosin Y (2.5 – 10 μM). Gels were then exposed through a patterned chrome photomask (Photo Sciences, Inc.) to collimated visible light (λ = 490 – 650 nm, 10 mW cm^{-2}, 0 – 2 min), achieved with an Acticure (EXFO) high pressure mercury lamp equipped with an internal bandpass filter (350 – 650 nm) and an external 490 nm longpass filter (Edmund Optics). Alternatively, multiphoton-based techniques were used to achieve 3D photocoupling, where selected regions of interest (x-y, ROI) at 1 μm z-plane increments within the hydrogel were scanned with pulsed laser light (λ = 860 nm, power = 350 mW μm^{-2}, scan speed = 1.27 μsec μm^{-2}) on a 710 LSM NLO confocal microscope stage (Carl Zeiss) equipped with a 20x/0.8 Plan-Apochromat objective (NA = 1.0, Carl Zeiss). Here, user-defined ROIs in the x-y plane were scanned over a given range in the z dimension. By scanning different sets of ROIs for different z-locations within the gel, patterns containing variability in all three spatial dimensions were obtained. For helix generation shown in Figure 2b, two circular ROIs in the x-y plane were scanned every micron from z = 0 to z = -10 μm. The circles were each rotated about a fixed center point by a preset amount (360°/20 rotations = 18°) and the sample was then scanned from z = -10 μm to z = -20 μm. This process of scanning and circle shifting was repeated 50 times to generate a helix spanning 2.5 full rotations. Upon completion of the photocoupling process, unreacted patterning agent and photoinitiator were swollen into PBS as the sample was gently agitated on an orbital shaker (2 hours) to yield the biochemically-patterned hydrogel by photoaddition. Photolithographic photoremoval of the peptide was accomplished using UV light (λ = 365 nm, 5 – 20 mW cm^{-2}, 0 – 10 min) shown through a patterned chrome mask. The photoremoval process was controlled in 3D with

multiphoton-based techniques (λ = 740 nm, power = 670 mW μm^{-2}, scan speed = 1.27 $\mu sec\ \mu m^{-2}$). After UV light exposure, the sample was gently agitated in PBS on an orbital shaker (2 hours) to remove the released peptide and yield the newly-patterned hydrogel. For both the photocoupling and photoremoval processes, the reaction was visualized with confocal microscopy and found to be confined to only those volumes within the material that were exposed to light. The resolution obtained for multiphoton-based patterning is ~1 μm in the x-y plane and ~3-5 μm in the z plane, which is similar to that obtained for multiphoton imaging methods.

Biochemical Gradient Generation by Photorelease: Prepatterned hydrogels containing ~1 mM of Ac-C-[PL]-RGDSK(AF_{488})-NH_2 were exposed to collimated UV light (365 nm, 10 mW cm^{-2}) while a unidirectionally-moving photomask covered the sample at a fixed rate.[19,22] As the rate of coverage was easily varied (1.6, 0.8, 0.4 mm min^{-1}), unique and well-defined gradients of light were readily generated and imposed on the hydrogel. After UV light exposure, the sample was gently agitated in PBS on an orbital shaker (2 hours) to remove the released peptide and yield the gradiant-patterned hydrogel.

Quantification of Patterning Concentration: The level of peptide incorporation (Ac-C-[PL]-RGDSK(AF_{488})-NH_2) was quantified by comparing the patterned fluorescence from the AF_{488} against that from gels swollen with known uncoupled free concentrations of the same patterning agent. As expected, the fluorescence of the swollen gels increased linearly with peptide concentration and provided a standard curve for relating fluorescence intensity with concentration. Though this method required a standard curve to be generated for each peptide of interest, it only required a small subset of the peptide population to be fluorescently labeled (<1%).

Cell Culture: NIH 3T3s were cultured at 37 ^{o}C and 5% CO_2 in high-glucose Dulbecco's modified Eagle's medium (Gibco) containing 10% fetal bovine serum (Invitrogen), 1% penicillin/streptomycin (Gibco), 0.2% fungizone, and 0.4% gentamicin.

Cell Seeding: Prepatterned click hydrogels attached to azide-functionalized glass slides were placed in a non-TC-treated 6-well cell culture plate (Corning) and ~80 x 10^3 cells (NIH 3T3s) suspended in 4 mL of fresh media were added to each well (surface area of well bottom = 9.6 cm^2) resulting in a seeding density

of 8 x 10^3 cells cm^{-2}. 4 hours after seeding, the gels were gently rinsed with fresh media to remove non-adherant cells.

Cell Staining: 24 hours after seeding, or 4 hours after releasing RGD, cells were fixed in formalin for 1 h, followed by cell permeabilization with 0.5% Triton® X-100 (Fisher) in PBS for 45 min. The gels were blocked with 3% bovine serum albumin (BSA, Sigma) in PBS for 30 min and rinsed with PBS. F-actin was visualized using Alexa Fluor® 488 Phalloidin Conjugate (5 U/mL, Invitrogen), while nuclei were stained with DAPI (500 nM, Invitrogen), each of which was applied for 1 h. The samples were washed with PBS prior to visualization with confocal microscopy.
